# Association between Prognostic Nutritional Index and myelosuppression in gastric cancer patients undergoing chemotherapy: a retrospective cohort study

**DOI:** 10.3389/fnut.2025.1605421

**Published:** 2025-10-07

**Authors:** Kexia Chen, Lifang Xiao, Bing Xiao, Longwu Zeng, Weiming Liu, Yafen Guo, Xueqing Zhang

**Affiliations:** ^1^Department of Oncology, Changde Hospital, Xiangya School of Medicine, Central South University (The First People's Hospital of Changde City), Changde, China; ^2^Department of Nursing, Changde Hospital, Xiangya School of Medicine, Central South University (The First People's Hospital of Changde City), Changde, China; ^3^Department of Pathology, The Affiliated Cancer Hospital of Xiangya School of Medicine, Central South University (Hunan Cancer Hospital), Changsha, China

**Keywords:** Prognostic Nutritional Index, gastric cancer, chemotherapy, myelosuppression, predictive biomarker, nutritional status

## Abstract

**Objective:**

To investigate the association between Prognostic Nutritional Index (PNI) and chemotherapy-induced myelosuppression in gastric cancer patients.

**Methods:**

This retrospective cohort study analyzed 562 gastric cancer patients receiving chemotherapy at two Chinese medical centers from January 2022 to December 2024. The exposure variable was PNI, calculated from serum albumin and lymphocyte count. The primary outcome was myelosuppression after the first chemotherapy cycle, defined according to CTCAE 5.0 criteria. Multiple logistic regression models adjusted for demographics, health status, tumor characteristics, treatment factors, and laboratory parameters.

**Results:**

Myelosuppression occurred in 75.1% of patients. After full adjustment, each one-unit increase in PNI reduced myelosuppression risk by 13% (OR = 0.87, 95%CI: 0.79–0.96, *p* = 0.004). Patients with PNI ≤ 48 had a significantly higher risk of myelosuppression (OR = 14.50, 95%CI: 4.93–42.65, *p* < 0.001). Significant effect modification was observed by sex (interaction *p* < 0.001), with stronger protective effects in males (OR = 0.71, 95%CI: 0.60–0.84).

**Conclusion:**

PNI is an independent predictor of chemotherapy-induced myelosuppression in gastric cancer patients, with a threshold of ≤48 identifying high-risk individuals. This readily available biomarker may guide personalized preventive strategies, particularly for male patients.

## 1 Introduction

Gastric cancer remains one of the most prevalent malignancies globally, with significant morbidity and mortality. According to the International Agency for Research on Cancer (IARC), approximately 1,089,000 new cases of gastric cancer were diagnosed worldwide in 2020, with 769,000 associated deaths (1, 2). China faces a significant gastric cancer burden, with 358,672 new cases in 2022 (7.4% of all cancer cases in China), making it the fifth most common cancer overall. The age-standardized incidence rate is 13.7 per 100,000 population. Gastric cancer ranks third in cancer mortality in China, causing 260,372 deaths (10.1% of all cancer deaths) (3, 4). Despite therapeutic advances, the 5-year survival rates for advanced gastric cancer remain between 5 and 20% (5).

Systemic therapies for GC, including chemotherapy, targeted therapy, and immunotherapy, have evolved significantly in the past few years, with chemotherapy as the standard treatment (6). However, chemotherapy-induced myelosuppression represents one of the most common and potentially serious adverse effects, which can lead to treatment interruptions, dose reductions, or even life-threatening complications (7). Myelosuppression results in a serious complication during tumor chemotherapy or radiation therapy. This condition significantly impairs the patient’s hematopoietic function and disrupts the balance within the bone marrow microenvironment. Such pathological changes may further trigger a series of dangerous clinical issues, including infections due to compromised immune defense, hemorrhagic disorders, anemia-related symptoms, and even multiple organ dysfunction in severe cases (8). Studies indicate that approximately 79% of cancer patients experience myelosuppression during chemotherapy, with treatment modifications required in approximately 64% of cases due to this complication (9). In gastric cancer specifically, the incidence of grade ≥2 myelosuppression during first-line chemotherapy has been reported at 26.5% in recent studies (10). The ability to identify patients at high risk for chemotherapy-induced myelosuppression could significantly improve clinical decision-making, enabling personalized treatment approaches and preventive interventions.

The prognostic nutrition index (PNI) is a reliable indicator for predicting the prognosis of various cancers after treatment, especially in gastric cancer (11, 12). PNI is calculated as albumin (g/L) + 5 × peripheral lymphocyte count (10^9^/L), offering a comprehensive evaluation that reflects both nutritional status (serum albumin) and immune function (lymphocyte count) (13). In gastric cancer specifically, low PNI values have been consistently associated with more aggressive disease characteristics, increased postoperative complications, and poorer overall survival (14, 15). Recent studies by Hirahara et al. confirmed that patients with low PNI exhibited significantly worse cancer-specific survival and higher rates of postoperative complications following gastrectomy (16). Additionally, another study by Park et al. demonstrated that both preoperative low PNI values and decreased PNI before/after surgery were associated with poor prognosis in a cohort of 1,868 gastric cancer patients (11). The predictive utility of PNI extends beyond surgical outcomes, with recent evidence suggesting its potential value in predicting response to immunotherapy in advanced gastric cancer patients (17, 18).

The relationship between nutritional status, immune function, and chemotherapy tolerance has garnered increasing attention in oncological research. Recent evidence suggests a potential link between PNI and chemotherapy-induced myelosuppression, particularly in gastric cancer patients. A retrospective study of 102 stage IV gastric cancer patients receiving first-line chemotherapy found that patients with low PNI values experienced significantly higher incidences of grade ≥2 myelosuppression after the first cycle of chemotherapy (*p* = 0.001) (10). Furthermore, high PNI values were associated with higher chemotherapy completion rates (*p* = 0.001), suggesting better treatment tolerance (10). In contrast, low PNI values indicate malnutrition and compromised immune function, potentially rendering patients more susceptible to chemotherapy toxicity (10). The biological rationale for this association may lie in the fact that PNI incorporates lymphocyte count, which reflects bone marrow function and immune system status (19).

Based on these findings, this study aims to explore the association between PNI and chemotherapy-induced myelosuppression in gastric cancer patients through a retrospective cohort analysis. By controlling for potential confounding factors, we will evaluate PNI as an independent predictor of myelosuppression risk following chemotherapy. Establishing this simple and cost-effective predictive biomarker has significant clinical implications, facilitating personalized risk assessment and preventive strategies, including chemotherapy regimen modifications, and enhanced monitoring protocols. Furthermore, interventions targeting nutritional status and immune function may reduce myelosuppression incidence and severity, thereby improving treatment adherence and quality of life. This research will provide evidence-based insights into the field of nutritional oncology and advance individualized treatment approaches for gastric cancer patients.

## 2 Method

### 2.1 Study design and participants

Between January 2022 and December 2024, we conducted a retrospective cohort study at two medical centers in China: Hunan Cancer Hospital and The First People’s Hospital of Changde. A total of 562 gastric cancer patients undergoing chemotherapy were included. All patients were diagnosed with gastric carcinoma confirmed by pathological biopsy. The inclusion criteria were: (1) histologically confirmed diagnosis of gastric carcinoma; (2) initial chemotherapy and complete chemotherapy; (3) survival time ≥ 3 months; (4) age ≥ 18 years; (5) chemotherapy dose was either standard or low dose; (6) complete and accessible clinical data. We excluded patients with the following conditions: (1) other malignant tumors or history of infection; (2) insufficiency of vital organs, including heart, liver, kidney, and brain; (3) inability to cooperate or incomplete clinical data; (4) history of myelosuppression or prophylactic use of leukocyte-stimulating agents prior to chemotherapy; and (5) history of radiotherapy. Data were collected from electronic medical records using standardized extraction forms. Two trained researchers independently extracted the data, with discrepancies resolved by a senior investigator.

### 2.2 Study indicators

#### 2.2.1 Exposure

The exposure variable was the Prognostic Nutritional Index (PNI), calculated using Sun’s (13) formula: PNI = albumin (g/L) + 5 × peripheral lymphocyte count (10^9^/L). Serum albumin and lymphocyte count were uniformly collected across all patients within 3 days before chemotherapy initiation regardless of surgery status.

#### 2.2.2 Outcome

The primary outcome was the occurrence of chemotherapy-induced myelosuppression after completing the first cycle of chemotherapy. According to the National Cancer Institute Common Terminology Criteria for Adverse Events (CTCAE 5.0) (20), myelosuppression was determined as satisfying one of the following criteria: (1) white blood cells (WBC) < 4 × 10^9^/L, (2) neutrophils <2 × 10^9^/L, (3) platelets (PLT) < 100 × 10^9^/L, and (4) hemoglobin (Hb) < 110 g/L. Myelosuppression was assessed by two experienced oncologists independently, and any discrepancies were resolved through discussion until consensus was reached.

#### 2.2.3 Covariates

To control for potential confounding effects, we included the following covariates, most collected at baseline before chemotherapy: (1) demographic characteristics, including age, gender, and education level; (2) general health conditions, including body mass index (BMI, calculated as a person’s body weight in kilograms divided by the square of their height in meters), karnofsky performance status score (KPS, a scale ranging from 0 to 100, with higher scores indicating better functional status and ability to perform daily activities), history of chronic diseases (e.g., hypertension, diabetes, and coronary heart disease), history of smoking, and history of alcohol use; (3) tumor-specific characteristics, including tumor stage, lymph node metastasis, and whether surgery; (4) chemotherapy-related factors, including chemotherapy regimen, chemotherapy dosage, additional nutritional supplements after chemotherapy, and length of hospitalization during chemotherapy; (5) laboratory parameters, which were obtained from initial blood tests performed within 3 days before chemotherapy initiation in gastric cancer patients. They included carcinoembryonic antigen (CEA, ng/L), carbohydrate antigen 19–9 (CA199, U/mL), white blood cell count (WBC, 10^9^/L), red blood cell count (RBC, 10^12^/L), neutrophil (10^9^/L), monocyte (10^9^/L), platelet (10^9^/L), hemoglobin (Hb, g/L), alanine aminotransferase (ALT, u/L), aspartate aminotransferase (AST, u/L), total bilirubin (Tbil, μmol/L), globulin (GLB, g/L), and creatine (μmol/L). These covariates were selected based on previous research indicating their potential associations with myelosuppression or PNI.

### 2.3 Ethics statement

This study protocol was approved by the Ethics Committees of the First People’s Hospital of Changde (approval number: 2024-066-01). As this was a retrospective study with all patient data de-identified prior to analysis, ensuring patient privacy and data confidentiality, the requirement for informed consent was waived in accordance with the principles of the Declaration of Helsinki. All research personnel strictly adhered to data protection regulations and signed confidentiality agreements. All collected data were used exclusively for the current research purposes, and results will be published only in aggregate form without any personally identifiable information.

### 2.4 Statistical analysis

All analyses were performed using R statistical software,^1^ with two-sided *p* values <0.05 considered statistically significant. Continuous variables were presented as means ± standard deviations (normal distribution) or medians and interquartile ranges (non-normal distribution), while categorical variables were expressed as frequencies and percentages. Differences between groups were compared using the *χ*^2^ test (categorical variables), Student’s t-test (normal distribution), or the Mann–Whitney U test (non-normal distribution).

The PNI cutoff was determined using receiver operating characteristic (ROC) curve analysis and the Youden index. ROC curves graphically represent the trade-off between the actual positive rate (sensitivity) and the false positive rate (1-specificity) across various cutoff values, while the area under the curve (AUC) quantifies the overall discriminative power (21). The Youden index (J) was used to identify the optimal cutoff point on the ROC curve, which was calculated as J = Sensitivity + Specificity − 1 (21) Internal validation was performed using bootstrapping method, which involved repeatedly resampling the original dataset for 500 times to assess performance variability and obtain more accurate estimates (21). In addition, decision curve analysis (DCA) was performed to assess the clinical utility of PIN by considering the potential benefits and harms of different clinical actions at various threshold probabilities (22).

To investigate the association between PNI and myelosuppression, we employed univariate and multivariate linear regression models with three levels of adjustment: model I with no covariate adjustment; model II adjusted for age and gender only; and Model III further adjusted for other covariates presented in Table 1. This progressive adjustment strategy aimed to evaluate the effect size trends of PNI under different adjustment strategies to verify the robustness of our findings. To ensure analytical robustness, we conducted sensitivity analysis by converting PNI into a categorical variable and calculating *p* for trend, verifying the continuous variable results, and examining potential non-linearity.

**Table 1:** Baseline characteristics stratified by the presence or absence of myelosuppression (*n* = 562).

		Total (*n* = 562)	No myelosuppression (*n* = 140)	Myelosuppression (*n* = 422)	*p*-value^&^
Exposure					
PNI, Median (IQR)		43.86 (5.71)	46.98 (6.37)	42.83 (5.08)	<0.001
ALB (g/L), Mean (SD)		37.48 (4.76)	39.54 (4.88)	36.79 (4.53)	<0.001
Lymphocyte (10^9^/L), Median (IQR)		1.21 (0.94–1.46)	1.31 (1.14–1.78)	1.17 (0.90–1.42)	<0.001
Demographic characteristics					
Age (years), Mean (SD)		52.99 (10.37)	50.71 (8.76)	53.74 (10.76)	0.003
Gender, *n* (%)	Female	244 (43.42%)	74 (52.86%)	170 (40.28%)	0.009
	Male	318 (56.58%)	66 (47.14%)	252 (59.72%)	
Education level, *n* (%)	Associate degree and below	329 (58.54%)	76 (54.29%)	253 (59.95%)	0.415
	Bachelor’s degree	149 (26.51%)	39 (27.86%)	110 (26.07%)	
	Master’s degree or above	84 (14.95%)	25 (17.86%)	59 (13.98%)	
General health conditions					
BMI (kg/m^2^), Mean (SD)		21.50 (3.11)	21.53 (2.84)	21.49 (3.19)	0.906
KPS, Mean (SD)		84.41 (7.68)	84.29 (8.91)	84.45 (7.23)	0.821
History of chronic diseases, *n* (%)	No	260 (46.26%)	52 (37.14%)	208 (49.29%)	0.004
	Other	66 (11.74%)	10 (7.14%)	56 (13.27%)	
	Hypertension	88 (15.66%)	28 (20.00%)	60 (14.22%)	
	Diabetes	78 (13.88%)	28 (20.00%)	50 (11.85%)	
	Coronary heart disease	70 (12.46%)	22 (15.71%)	48 (11.37%)	
History of smoking, *n* (%)	No	311 (55.34%)	90 (64.29%)	221 (52.37%)	0.014
	Yes	251 (44.66%)	50 (35.71%)	201 (47.63%)	
History of alcohol use, *n* (%)	No	347 (61.74%)	115 (82.14%)	232 (54.98%)	<0.001
	Yes	215 (38.26%)	25 (17.86%)	190 (45.02%)	
Tumor-specific characteristics					
Tumor stage, *n* (%)	II	18 (3.20%)	6 (4.29%)	12 (2.84%)	0.080
	III	274 (48.75%)	78 (55.71%)	196 (46.45%)	
	IV	270 (48.04%)	56 (40.00%)	214 (50.71%)	
Lymph node metastasis, *n* (%)	No	164 (29.18%)	48 (34.29%)	116 (27.49%)	0.125
	Yes	398 (70.82%)	92 (65.71%)	306 (72.51%)	
Whether surgery, *n* (%)	No	222 (39.50%)	68 (48.57%)	154 (36.49%)	0.011
	Yes	340 (60.50%)	72 (51.43%)	268 (63.51%)	
Chemotherapy-related factors					
Chemotherapy regimen, *n* (%)	XELOX	108 (19.22%)	26 (18.57%)	82 (19.43%)	0.611
	SOX	116 (20.64%)	25 (17.86%)	91 (21.56%)	
	FOLFOX	103 (18.33%)	22 (15.71%)	81 (19.19%)	
	DOS	103 (18.33%)	27 (19.29%)	76 (18.01%)	
	FLOT	96 (17.08%)	29 (20.71%)	67 (15.88%)	
	Other	36 (6.41%)	11 (7.86%)	25 (5.92%)	
Dosage range, *n* (%)	Low	44 (7.83%)	8 (5.71%)	36 (8.53%)	0.282
	Normal	518 (92.17%)	132 (94.29%)	386 (91.47%)	
Additional nutritional supplements, *n* (%)	No	163 (29.00%)	41 (29.29%)	122 (28.91%)	0.029
	ONS	170 (30.25%)	55 (39.29%)	115 (27.25%)	
	ONS + TPN	139 (24.73%)	27 (19.29%)	112 (26.54%)	
	TPN	90 (16.01%)	17 (12.14%)	73 (17.30%)	
Length of hospital (day), Mean (SD)		7.00 (5.00–10.00)	7.00 (5.00–8.00)	6.00 (5.00–11.00)	0.250
Laboratory parameters					
CEA (ng/L), Median (IQR)		3.05 (1.75–6.73)	2.86 (1.57–7.05)	3.08 (1.82–6.55)	0.553
CA199 (U/mL), Median (IQR)		13.75 (5.64–61.33)	12.18 (6.42–27.36)	14.46 (5.20–66.89)	0.823
WBC (10^9^/L), Median (IQR)		4.13 (3.21–5.53)	5.47 (4.62–6.50)	3.59 (2.97–4.87)	<0.001
RBC (10^12^/L), Median (IQR)		3.70 (3.26–4.07)	4.07 (3.82–4.32)	3.55 (3.10–3.96)	<0.001
Neutrophil (10^9^/L), Median (IQR)		2.23 (1.56–3.57)	3.31 (2.69–4.65)	1.89 (1.37–3.03)	<0.001
Monocyte (10^9^/L), Median (IQR)		0.35 (0.28–0.47)	0.35 (0.30–0.57)	0.35 (0.26–0.46)	0.009
Platelet (10^9^/L), Median (IQR)		177.00 (134.75–228.00)	189.00 (159.00–268.00)	171.00 (123.50–216.00)	<0.001
Hb (g/dL), Mean (SD)		104.94 (18.78)	122.49 (10.77)	99.20 (17.22)	<0.001
ALT (u/L), Median (IQR)		18.50 (12.00–30.00)	17.00 (13.00–29.00)	20.00 (12.00–31.75)	0.174
AST (u/L), Median (IQR)		25.00 (20.00–37.08)	21.00 (19.00–30.00)	25.00 (20.00–39.00)	0.002
TBil (U/mL), Mean (SD)		9.22 (3.42)	9.68 (3.56)	9.07 (3.37)	0.067
GLB (g/L), Mean (SD)		25.52 (4.14)	26.60 (4.60)	25.17 (3.92)	<0.001
Creatine (μmol/L), Median (IQR)		60.00 (48.00–68.00)	61.50 (49.00–71.00)	59.00 (48.00–67.00)	0.038

SD, standard deviation; IQR, interquartile range; BMI, Body Mass Index; KPS, Karnofsky Performance Status; XELOX, Capecitabine + Oxaliplatin; SOX, S-1 + Oxaliplatin; FOLFOX, Folinic acid + Fluorouracil + Oxaliplatin; DOS, Docetaxel + Oxaliplatin + S-1; FLOT, Fluorouracil + Leucovorin + Oxaliplatin + Docetaxel; Other, Other chemotherapy regimens; ONS, Oral Nutritional Supplements; TPN, Total Parenteral Nutrition; CEA, carcinoembryonic antigen; CA199, carbohydrate antigen 19–9; WBC, White Blood Cell count; RBC, Red Blood Cell count; Hb, Hemoglobin; ALT, Alanine Aminotransferase; AST, Aspartate Aminotransferase; ALB, Albumin; GLB, Globulin; TBil, Total Bilirubin; PNI, Prognostic Nutritional Index. ^&^*p*-value: Student’s t-test for continuous variables with normal distribution, Mann–Whitney U-tests for continuous variables with non-normal distribution; Chi-square test for categorical variables.

## 3 Results

### 3.1 Baseline characteristics by myelosuppression status

**Figure 1:**
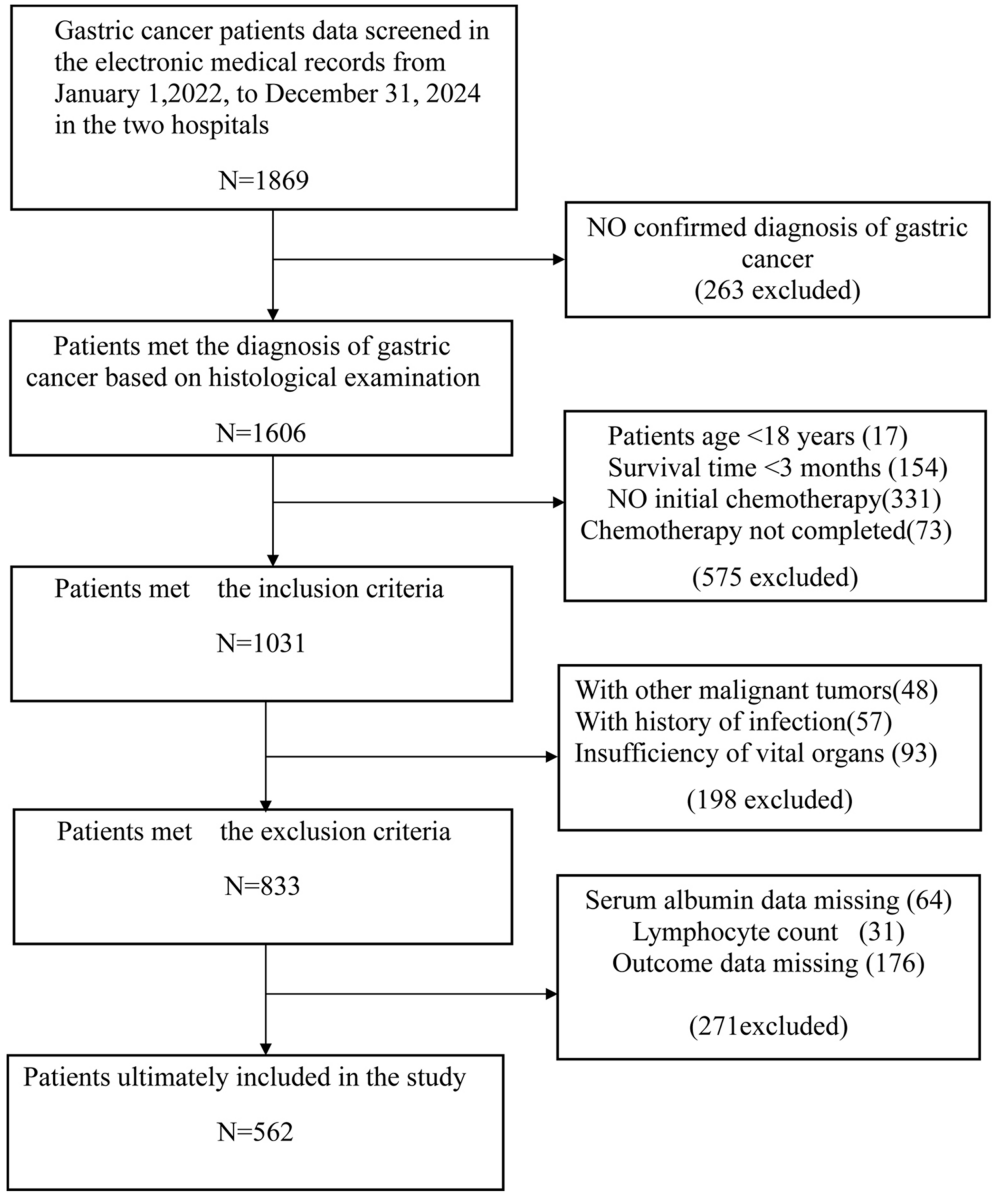
Flowchart of subject screening. Flowchart illustrating inclusion and exclusion criteria.

Overall, 562 subjects were included in the final analysis (Figure 1), among whom 422 developed myelosuppression (75.1%). Table 1 shows the comparison of sample characteristics between those with and without myelosuppression. Compared to the no-myelosuppression group, the myelosuppression group were older (53.74 vs. 50.71 years, *p* = 0.003), more likely to be male (59.72% vs. 47.14%, *p* = 0.009), and had higher rates of alcohol consumption (45.02% vs. 17.86%, *p* < 0.001) and smoking (47.63% vs. 35.71%, *p* = 0.014). The myelosuppression group exhibited lower levels of laboratory parameters, including neutrophil (1.89 vs. 3.31 × 10^9^/L), lymphocyte (1.17 vs. 1.31 × 10^9^/L), platelet counts (171.00 vs. 189.00 × 10^9^/L), hemoglobin (99.20 vs. 122.49 g/L), and albumin (36.78 vs. 39.54 g/L) (all *p* < 0.001). The myelosuppression group also had a higher utilization rate of additional nutritional supplements post-chemotherapy (71.09% vs. 70.71%, *p* = 0.029). Notably, PNI was substantially lower in patients experiencing myelosuppression (42.83 vs. 46.98, *p* < 0.001). In addition, a cutoff value of 48 (sensitivity: 0.876, specificity: 0.515, AUC: 0.729) was determined for PNI based on ROC and DCA. The ROC maximizes the combined sensitivity and specificity, while the DCA confirms the clinical utility of the cutoff. Details are shown in Supplementary Figures 1, 2 and Supplementary Table S1.

### 3.2 Univariate and multivariate analysis

Table 2 shows the univariate and multivariate analysis results to identify independent predictors for myelosuppression. Univariate analysis showed significant associations between myelosuppression and age, smoking, surgery, albumin, and chronic comorbidities. Multivariate analysis revealed alcohol consumption (OR = 4.35, 95% CI: 1.72–10.97) and prolonged hospitalization (OR = 1.48, 95% CI: 1.27–1.72) as independent risk factors for myelosuppression. In contrast, baseline neutrophil count (OR = 0.46 per 10^9^/L, 95% CI 0.35–0.61), lymphocyte count (OR = 0.45, 95% CI 0.24–0.84), hemoglobin (OR = 0.85 per g/L, 95% CI 0.82–0.89), and PNI (OR = 0.85 per unit, 95% CI 0.75–0.97) demonstrated robust protective effects.

**Table 2:** Univariate and multivariate analysis of myelosuppression.

		Mean (SD)/N (%)	Univariate		Multivariate	
			OR (95% CI)	*p*-value	OR (95% CI)	P-value
Exposure						
PNI, Median (IQR)		43.86 (5.72)	0.87 (0.83, 0.90)	<0.001	0.85 (0.75, 0.97)	0.012
ALB (g/L), Mean (SD)		37.48 (4.76)	0.87 (0.83, 0.91)	<0.001	1.09 (0.93, 1.28)	0.276
Lymphocyte (10^9^/L), Median (IQR)		1.28 (0.54)	0.38 (0.26, 0.54)	<0.001	0.45 (0.24, 0.84)	0.012
Demographic characters						
Age (years), Mean (SD)		52.99 (10.37)	1.03 (1.01, 1.05)	0.003	0.99 (0.95, 1.03)	0.719
Gender, *n* (%)	Female	244 (43.42%)	Ref		Ref	
	Male	318 (56.58%)	1.66 (1.13, 2.44)	0.010	1.43 (0.59, 3.49)	0.427
Education Status, *n* (%)	Associate degree and below	329 (58.54%)	Ref			
	Bachelor’s degree	149 (26.51%)	0.85 (0.54, 1.32)	0.467		
	Master’s degree or above	84 (14.95%)	0.71 (0.42, 1.21)	0.206		
General health conditions						
BMI (kg/m^2^), Mean (SD)		21.50 (3.11)	1.00 (0.94, 1.06)	0.906		
KPS, Mean (SD)		84.41 (7.68)	1.00 (0.98, 1.03)	0.821		
History of chronic diseases, *n* (%)	No	260 (46.26%)	Ref		Ref	
	Other	66 (11.74%)	1.40 (0.67, 2.93)	0.372	5.03 (0.73, 34.54)	0.101
	Hypertension	88 (15.66%)	0.54 (0.31, 0.92)	0.024	0.93 (0.30, 2.84)	0.892
	Diabetes	78 (13.88%)	0.45 (0.26, 0.78)	0.004	1.31 (0.41, 4.25)	0.649
	Coronary heart disease	70 (12.46%)	0.55 (0.30, 0.98)	0.044	1.06 (0.36, 3.06)	0.920
History of smoking, *n* (%)	No	311 (55.34%)	Ref		Ref	
	Yes	251 (44.66%)	1.64 (1.10, 2.43)	0.014	2.17 (0.92, 5.10)	0.075
History of alcohol use, *n* (%)	No	347 (61.74%)	Ref		Ref	
	Yes	215 (38.26%)	3.77 (2.35, 6.05)	<0.001	4.35 (1.72, 10.97)	0.002
Tumor-specific characteristics						
Tumor stage, *n* (%)	II	18 (3.20%)	Ref			
	III	274 (48.75%)	1.26 (0.46, 3.47)	0.659		
	IV	270 (48.04%)	1.91 (0.69, 5.32)	0.215		
Lymph node metastasis, *n* (%)	No	164 (29.18%)	Ref			
	Yes	398 (70.82%)	1.38 (0.91, 2.07)	0.126		
Whether surgery, *n* (%)	No	222 (39.50%)	Ref		Ref	
	Yes	340 (60.50%)	1.64 (1.12, 2.42)	0.012	0.46 (0.18, 1.20)	0.114
Chemotherapy-related factors						
Chemotherapy regimen, *n* (%)	XELOX	108 (19.22%)	Ref			
	SOX	116 (20.64%)	1.15 (0.62, 2.16)	0.653		
	FOLFOX	103 (18.33%)	1.17 (0.61, 2.23)	0.638		
	DOS	103 (18.33%)	0.89 (0.48, 1.66)	0.720		
	FLOT	96 (17.08%)	0.73 (0.39, 1.36)	0.325		
	Other	36 (6.41%)	0.72 (0.31, 1.66)	0.442		
Dosage range, *n* (%)	Low	44 (7.83%)	Ref			
	Normal	518 (92.17%)	0.65 (0.29, 1.43)	0.286		
Additional nutritional supplements, *n* (%)	No	163 (29.00%)	Ref			
	ONS	170 (30.25%)	0.70 (0.44, 1.13)	0.148		
	ONS + TPN	139 (24.73%)	1.39 (0.80, 2.41)	0.236		
	TPN	90 (16.01%)	1.44 (0.76, 2.72)	0.258		
Length of hospital (day), mean (SD)		8.42 (5.69)	1.10 (1.04, 1.16)	<0.001	1.48 (1.27, 1.72)	<0.001
Laboratory parameters						
CEA (ng/L), Median (IQR)		23.39 (96.62)	1.00 (1.00, 1.01)	0.106		
CA199 (U/mL), Median (IQR)		164.89 (536.46)	1.00 (1.00, 1.01)	0.104		
WBC (10^9^/L), Median (IQR)		4.99 (6.69)	0.97 (0.94, 1.01)	0.116		
Neutrophil (10^9^/L), Median (IQR)		2.80 (1.89)	0.68 (0.61, 0.75)	<0.001	0.46 (0.35, 0.61)	<0.001
RBC (10^12^/L), Median (IQR)		4.14 (6.50)	1.00 (0.97, 1.04)	0.861		
Monocyte (10^9^/L), Median (IQR)		0.42 (0.51)	0.42 (0.17, 1.08)	0.072		
Platelet (10^9^/L), Median (IQR)		193.43 (89.09)	1.00 (0.99, 1.00)	<0.001	0.99 (0.99, 1.00)	0.001
Hb (g/dL), Mean (SD)	Hb, Mean (SD)	104.94 (18.78)	0.90 (0.88, 0.91)	<0.001	0.85 (0.82, 0.89)	<0.001
ALT (u/L), Median (IQR)	ALT, Mean (SD)	26.77 (27.67)	1.00 (0.99, 1.01)	0.725		
AST (u/L), Median (IQR)	AST, Mean (SD)	33.92 (42.96)	1.00 (1.00, 1.01)	0.668		
TBil (U/mL), Mean (SD)	TBil, Mean (SD)	9.22 (3.42)	0.95 (0.90, 1.00)	0.068		
GLB (g/L), Mean (SD)	GLB, Mean (SD)	25.52 (4.14)	0.92 (0.88, 0.97)	<0.001	0.95 (0.87, 1.04)	0.298
Creatine (μmol/L), Median (IQR)	Creatine, Mean (SD)	60.47 (23.05)	0.99 (0.98, 1.00)	0.018	0.95 (0.92, 0.98)	0.003

SD, standard deviation; OR, odds ratio; CI, confidence interval; Ref, reference group for statistical analysis; BMI, Body Mass Index; KPS, Karnofsky Performance Status; XELOX, Capecitabine + Oxaliplatin; SOX, S-1 + Oxaliplatin; FOLFOX, Folinic acid + Fluorouracil + Oxaliplatin; DOS, Docetaxel + Oxaliplatin + S-1; FLOT, Fluorouracil + Leucovorin + Oxaliplatin + Docetaxel; Other, Other chemotherapy regimens; ONS, Oral Nutritional Supplements; TPN, Total Parenteral Nutrition; CEA, carcinoembryonic antigen; CA199, carbohydrate antigen 19–9; WBC, White Blood Cell count; RBC, Red Blood Cell count; Hb, Hemoglobin; ALT, Alanine Aminotransferase; AST, Aspartate Aminotransferase; ALB, Albumin; GLB, Globulin; TBil, Total Bilirubin; PNI, Prognostic Nutritional Index.

### 3.3 Subgroup analysis

Table 3 shows the subgroup analyses of the association between PNI and myelosuppression across various demographic and clinical characteristics. The results revealed significant heterogeneity in the protective effect of PNI against myelosuppression. A marked sex-based dichotomy was observed (interaction *p* < 0.001), with each one-unit PNI increase conferring substantial protection in males (OR = 0.71, 95% CI 0.60–0.84, *p* < 0.001) but no effect in females (OR = 1.12, 95% CI 0.92–1.38, *p* = 0.260). The protective effect remained consistent across other indicators, with no significant interactions by age, surgery, smoking history, alcohol history, lymph node metastasis, chemotherapy dosage, tumor stage, white blood cell count, hemoglobin, or platelet count.

**Table 3:** Effect size of PNI (as a continuous variable) on myelosuppression across subgroups.

Characteristics	No. of participants	Myelosuppression		
		OR (95% CI)	*p*	*p* for interaction
Age (years), *n* (%)				0.852
<60	394	0.84 (0.74, 0.94)	0.003	
≥60	168	0.85 (0.70, 1.04)	0.120	
Gender, *n* (%)				<0.001
Female	244	1.12 (0.92, 1.38)	0.263	
Male	318	0.71 (0.60, 0.84)	<0.001	
Whether surgery, *n* (%)				0.117
No	222	0.93 (0.77, 1.13)	0.463	
Yes	340	0.78 (0.68, 0.88)	<0.001	
History of smoking, *n* (%)				0.986
No	311	0.89 (0.78, 1.00)	0.057	
Yes	251	0.89 (0.77, 1.02)	0.092	
History of alcohol use, *n* (%)				0.589
No	347	0.89 (0.79, 0.99)	0.032	
Yes	215	0.83 (0.67, 1.04)	0.101	
Lymph node metastasis, *n* (%)				0.109
No	164	0.95 (0.79, 1.13)	0.553	
Yes	398	0.80 (0.70, 0.90)	<0.001	
Tumor stage, *n* (%)				0.198
II	18	0.26 (0.80, 1.03)	0.996	
III	274	0.70 (0.53, 0.92)	0.101	
IV	270	0.90 (0.78, 1.05)	0.184	
PLT classification (10^9^/L), *n* (%)				0.502
100–300	444	0.87 (0.79, 0.95)	0.003	
<100	52	0.95 (0.05, 0.99)	0.100	
>300	66	0.72 (0.52, 1.00)	0.049	
WBC classification (10^9^/L), *n* (%)				0.213
4–10	277	0.79 (0.69, 0.91)	<0.001	
<4	267	1.11 (0.77, 1.60)	0.571	
>10	18	1.01 (0.00, 1.098)	1.000	
Hb classification (g/dL), *n* (%)				0.112
120–160^*^, 110–150^#^	182	1.40 (0.52, 3.77)	0.505	
<120^*^, <110^#^	380	0.81 (0.72, 0.91)	<0.001	

PNI, Prognostic Nutritional Index; OR, odds ratio; CI, confidence interval; PLT, Platelet count; WBC, White Blood Cell count; Hb, Hemoglobin.

^*^indicates male subjects, ^#^indicates female subjects.

### 3.4 Independent association between PNI and myelosuppression

As shown in Table 4, multivariate logistic regression revealed PNI as a robust independent predictor of chemotherapy-induced myelosuppression. When analyzed as a continuous variable, each one-unit PNI increase conferred significant protection against myelosuppression in unadjusted (OR = 0.87, 95% CI 0.83–0.90, *p* < 0.001), demographically-adjusted (OR = 0.87, 95% CI 0.83–0.90, *p* < 0.001), and fully-adjusted models incorporating clinical and laboratory parameters (OR = 0.87, 95% CI 0.79–0.96, *p* = 0.004). Categorically, patients with PNI ≤ 48 exhibited markedly elevated myelosuppression risk compared to patients with PNI > 48 across all models (unadjusted: OR = 7.12, 95% CI 4.58–11.05; demographically-adjusted: OR = 7.36, 95% CI 4.67–11.61; fully-adjusted: OR = 14.50, 95% CI 4.93–42.65; all *p* < 0.001). Significant trend tests confirmed a dose–response relationship between nutritional status and myelosuppression risk.

**Table 4:** Multivariate logistic regression analysis of PNI predicting myelosuppression.

Logistic regression model	Myelosuppression					
	Model I		Model II		Model III	
	OR (95% CI)	*p*-value	OR (95% CI)	*p*-value	OR (95% CI)	*p*-value
PNI as a continuous variable	0.87 (0.83, 0.90)	<0.001	0.87 (0.83, 0.90)	<0.001	0.87 (0.79, 0.96)	0.004
PNI as a categorical variable						
PNI (>48.025)	Ref		Ref		Ref	
PNI (≤48.025)	7.12 (4.58, 11.05)	<0.001	7.36 (4.67, 11.61)	<0.001	14.50 (4.93, 42.65)	<0.001
*p* for trend		<0.001		<0.001		0.008

Model I: unadjusted.

Model II: adjust for: Age, Gender.

Model III: adjust for: Age, Gender, History of smoking, History of alcohol use, Length of Hospital days, History of chronic diseases, Karnofsky Performance Status (KPS), Body Mass Index (BMI), Tumor stage, Lymph node metastasis, Whether surgery, Dosage range, Chemotherapy regimen, Additional nutritional supplements, Creatine, Monocyte, Neutrophil, Hemoglobin, Platelet, White Blood Cell count, Red Blood Cell count.

PNI, Prognostic Nutritional Index; Ref, reference group for statistical analysis; OR, odds ratio; CI, confidence interval.

## 4 Discussion

This retrospective study included 562 gastric cancer patients undergoing chemotherapy across two centers (2022–2024) to examine the association between PNI and chemotherapy-induced myelosuppression. Our results demonstrated a significant inverse relationship between PNI and myelosuppression risk. Multivariate analysis showed that each one-unit increase in PNI reduced myelosuppression risk by 13% (OR = 0.87, 95%CI: 0.79–0.96, *p* = 0.004) and patients with PNI ≤ 48 had substantially higher risk of myelosuppression (OR = 14.05, 95%CI: 4.93–42.65, *p* < 0.001). This association remained robust after adjusting for multiple confounders.

Our findings closely align with Wei et al.’s prospective study of 102 stage IV gastric cancer patients, which demonstrated that lower PNI was associated with an increased risk of grade ≥2 myelosuppression, while higher PNI was associated with improved chemotherapy completion rates (10). Using intravital imaging techniques, Nakasone et al. (23) demonstrated that the tumor microenvironment critically contributed to drug response through the regulation of vascular permeability and immune cell infiltration. Liu et al.’s (24) study primarily focused on the predictive value of nutritional and inflammatory markers for survival in stage II–III gastric cancer patients. Their findings support the importance of nutritional status in chemotherapy tolerance for gastric cancer patients, consistent with our observed association between PNI and myelosuppression. This finding is also consistent with Chen et al.’s (25) observations in breast cancer patients, where they found significant associations between PNI and hematological toxicities, including anemia, leukopenia, and myelosuppression.

Through a large cohort and comprehensive confounder adjustment, we established PNI ≤ 48 as a clinical threshold for significantly elevated myelosuppression risk. This threshold provides clinicians with an accessible, cost-effective screening tool that can be readily incorporated into routine pretreatment evaluations. Unlike complex genetic markers or specialized testing, PNI utilizes standard laboratory parameters (serum albumin and lymphocyte count) that were routinely collected during care, facilitating immediate clinical implementation without additional resource burden (26–28). The clinical significance of our findings extends beyond mere statistical associations, offering a practical framework for personalized risk assessment and management of chemotherapy-induced myelosuppression in gastric cancer.

Our study highlights the central role of PNI as an integrated marker of nutritional, immunological, and hematopoietic function. This provides a cohesive framework for exploring diverse mechanisms involving inflammation, pharmacokinetics, and the bone marrow microenvironment (29, 30). Specifically, lower PNI indicates chronic inflammation and a weaker immune response, which can impact the bone marrow’s ability to produce immune cells effectively (31). Additionally, lower PNI suggests nutritional deficiencies, which can affect the metabolism and distribution of drugs within the body, altering pharmacokinetic profiles (32). Furthermore, lower PNI signifies an unhealthy bone marrow microenvironment, which can impact the differentiation and maturation of hematopoietic cells and the overall immune response (33). All these mechanisms will contribute to the development of myelosuppression. By focusing on PNI as a comprehensive biomarker that integrates various physiological aspects, our study offers critical insights into the interconnectedness of nutritional deficiencies, immune system dysfunction, and impaired bone marrow function.

The subgroup analyses revealed significant heterogeneity in the protective effect of PNI against chemotherapy-induced myelosuppression. Most notably, a marked sex-based dichotomy was observed (interaction *p* = 0.001), with each one-unit PNI increase conferring a significant 34% reduction in myelosuppression risk for male patients but no apparent protective effect in female patients. This may be related to factors such as females typically exhibiting stronger inflammatory responses and immune activity, as well as differences in sex hormones (estrogen, progesterone, and androgens) and genes associated with sex chromosomes between males and females (34). Our subgroup analyses reveal pronounced protective effects in male patients and those undergoing surgical intervention, enabling targeted nutritional support strategies, potentially sparing patients from chemotherapy-related complications while optimizing treatment efficacy. From a health policy perspective, PNI screening could reduce healthcare costs associated with myelosuppression management, including emergency department visits, hospitalization for infectious complications, and use of hematopoietic growth factors (35). Several studies propose that prechemotherapy nutritional optimization programs guided by PNI assessment could constitute a valuable adjunct to conventional supportive care protocols, with nutrition consultation and intervention becoming standard practice for patients with PNI below our identified threshold (36–38).

Our study offers several methodological strengths that enhance the reliability and clinical applicability of our findings. First, the multi-center design incorporating data from two medical institutions increases the generalizability of our results across different clinical settings. Second, our large sample of 562 gastric cancer patients provides robust statistical power for identifying clinically meaningful associations. Third, our comprehensive statistical approach—employing progressive adjustment models with increasing levels of covariate control—demonstrates the stability of our findings across different analytical frameworks. Fourth, our detailed subgroup analyses identified important effect modifications by sex and surgical status, providing clinically relevant insights for personalized risk assessment. Finally, our establishment of a specific PNI threshold (≤48) for elevated myelosuppression risk offers clinicians a practical, easily implemented screening parameter using standard laboratory measurements already collected during routine care.

Our study has several limitations. First, we excluded patients with specific conditions, such as those with comorbid malignancies, infections, and organ insufficiencies, which may limit the generalizability of the research findings. Future studies should validate our results in a broader, more diverse patient population. Second, the participants were recruited from two Chinese medical centers and may not represent patients in other healthcare settings. Future multicenter studies are needed to improve sample representativeness and external validity of our findings. Third, the retrospective, observational study design cannot determine causation between PNI and myelosuppression risk, and is subject to multiple biases, including selection bias, information bias, and confounding bias, which may affect the validity and reliability of the findings. Future prospective, longitudinal study designs, primarily randomized controlled trials (RCTs), are needed to obtain more reliable and robust evidence. Fourth, despite comprehensive statistical adjustments, we could not control for unmeasured confounders like dietary patterns, genetic factors affecting drug metabolism, and psychosocial variables. Future studies should consider adding these factors and employing various strategies like sensitivity analyses, instrumental variable analyses, and negative control methods to assess and mitigate the potential impact of unmeasured confounding.

Fifth, assessing myelosuppression after only the first chemotherapy cycle may not capture delayed or cumulative toxicities in subsequent treatment. Future studies with longer follow-ups and multiple assessments are needed to capture the full spectrum of chemotherapy-induced toxicities and track their dynamic evolution. Finally, the PNI cutoff of 48 demonstrates a high sensitivity of 87.6% but moderate specificity (51.5%), which requires careful consideration of the potential clinical implications of false positives. False-positive results can lead to unnecessary diagnostic procedures and interventions, causing significant psychological burdens, increased healthcare costs, and decreased trust in healthcare providers. Therefore, multi-stage confirmatory testing and careful clinical evaluation are needed to mitigate false positives. In addition, thorough patient education and shared decision-making are required when interpreting the results. Furthermore, future research should develop more specific tests and diagnostic tools to improve accuracy and reduce the burden of false positives.

## 5 Conclusion

Our study identifies PNI as an independent predictor of chemotherapy-induced myelosuppression in gastric cancer patients, with values ≤48 indicating high risk. Each one-unit PNI increase reduced myelosuppression risk by 13%, with stronger protective effects in males. This readily available, cost-effective biomarker could guide preemptive interventions and personalized chemotherapy management, advancing supportive care optimization in gastric cancer treatment.

## Data Availability

The datasets presented in this study can be found in online repositories. The names of the repository/repositories and accession number(s) can be found in the article/Supplementary material.

## Footnotes

^1^ http://www.R-project.org

## Supplementary material

SUPPLEMENTARY FIGURE 1 The smooth ROC curve of PNI for predicting chemotherapy-induced myelosuppression in gastric cancer patients, obtained using the bootstrapping method (resample: 500), with an area under the receiver operating characteristic curve (ROC).

SUPPLEMENTARY FIGURE 2 The DCA curve of PNI for the prediction of chemotherapy-induced myelosuppression in gastric cancer patients.

## References

[1] Sung H Ferlay J Siegel RL Laversanne M Soerjomataram I Jemal A. Global cancer statistics 2020: GLOBOCAN estimates of incidence and mortality worldwide for 36 cancers in 185 countries. CA Cancer J Clin. (2021) 71:209–49. doi: 10.3322/caac.21660, PMID: 33538338

[2] Morgan E Arnold M Camargo MC Gini A Kunzmann AT Matsuda T. The current and future incidence and mortality of gastric cancer in 185 countries, 2020-40: a population-based modelling study. EClinicalMedicine. (2022) 47:101404. doi: 10.1016/j.eclinm.2022.101404, PMID: 35497064 PMC9046108

[3] International Agency for Research on Cancer. Global cancer observatory: cancer today. Population fact sheets: China (2020). Available online at: https://gco.iarc.who.int/today (Accessed August 7, 2025).

[4] Cao W Chen HD Yu YW Li N Chen WQ. Changing profiles of cancer burden worldwide and in China: a secondary analysis of the global cancer statistics 2020. Chin Med J. (2021) 134:783–91. doi: 10.1097/CM9.0000000000001474, PMID: 33734139 PMC8104205

[5] Ilic M Ilic I. Epidemiology of stomach cancer. World J Gastroenterol. (2022) 28:1187–203. doi: 10.3748/wjg.v28.i12.1187, PMID: 35431510 PMC8968487

[6] Guan WL He Y Xu RH. Gastric cancer treatment: recent progress and future perspectives. J Hematol Oncol. (2023) 16:57. doi: 10.1186/s13045-023-01451-3, PMID: 37245017 PMC10225110

[7] Yao L Feng W Tao Y Tang C. Effect of Shengbai decoction on chemotherapy-induced myelosuppression and survival of gastric cancer patients after radical resection: a retrospective study. Med Sci Monit. (2022) 28:e935936. doi: 10.12659/MSM.935936, PMID: 35185148 PMC8876002

[8] Zhang T Zhou M Xiao D Liu Z Jiang Y Feng M. Myelosuppression alleviation and hematopoietic regeneration by tetrahedral-framework nucleic-acid nanostructures functionalized with osteogenic growth peptide. Adv Sci (Weinh). (2022) 9:e2202058. doi: 10.1002/advs.202202058, PMID: 35882625 PMC9507378

[9] Epstein RS Aapro MS Basu Roy UK Salimi T Krenitsky J Leone-Perkins ML. Patient burden and real-world management of chemotherapy-induced myelosuppression: results from an online survey of patients with solid tumors. Adv Ther. (2020) 37:3606–18. doi: 10.1007/s12325-020-01419-6, PMID: 32642965 PMC7340862

[10] Wei J Xiang W Wei H Hu X Lu Y Dong X. Impact of nutrition risk index, prognostic nutritional index and skeletal muscle index on early myelosuppression of first-line chemotherapy in stage IV gastric cancer patients. BMC Gastroenterol. (2024) 24:452. doi: 10.1186/s12876-024-03548-6, PMID: 39695992 PMC11654076

[11] Park SH Lee S Song JH Choi S Cho M Kwon IG. Prognostic significance of body mass index and prognostic nutritional index in stage II/III gastric cancer. Eur J Surg Oncol. (2020) 46:620–5. doi: 10.1016/j.ejso.2019.10.024 31668977

[12] Sasahara M Kanda M Ito S Mochizuki Y Teramoto H Ishigure K. The preoperative prognostic nutritional index predicts short-term and long-term outcomes of patients with stage II/III gastric cancer: analysis of a multi-institution dataset. Dig Surg. (2020) 37:135–44. doi: 10.1159/000497454 30840952

[13] Sun H Chen L Huang R Pan H Zuo Y Zhao R. Prognostic nutritional index for predicting the clinical outcomes of patients with gastric cancer who received immune checkpoint inhibitors. Front Nutr. (2022) 9:1038118. doi: 10.3389/fnut.2022.1038118, PMID: 36438745 PMC9686298

[14] Zhao Y Deng Y Peng J Sui Q Lin J Qiu M. Does the preoperative prognostic nutritional index predict survival in patients with liver metastases from colorectal cancer who underwent curative resection? J Cancer. (2018) 9:2167–74. doi: 10.7150/jca.25346, PMID: 29937936 PMC6010682

[15] Feng YW Wang HY Lin Q. Can the preoperative prognostic nutritional index be used as a postoperative predictor of gastric or gastroesophageal junction adenocarcinoma? World J Gastrointest Oncol. (2024) 16:2877–80. doi: 10.4251/wjgo.v16.i7.2877, PMID: 39072155 PMC11271764

[16] Hirahara N Tajima Y Fujii Y Kaji S Yamamoto T Hyakudomi R. Prognostic nutritional index as a predictor of survival in resectable gastric cancer patients with normal preoperative serum carcinoembryonic antigen levels: a propensity score matching analysis. BMC Cancer. (2018) 18:285. doi: 10.1186/s12885-018-4201-4, PMID: 29534689 PMC5850976

[17] Ding P Guo H Sun C Yang P Kim NH Tian Y. Combined systemic immune-inflammatory index (SII) and prognostic nutritional index (PNI) predicts chemotherapy response and prognosis in locally advanced gastric cancer patients receiving neoadjuvant chemotherapy with PD-1 antibody sintilimab and XELOX: a prospective study. BMC Gastroenterol. (2022) 22:121. doi: 10.1186/s12876-022-02199-9, PMID: 35287591 PMC8919583

[18] Pan Y Ma Y Dai G. The prognostic value of the prognostic nutritional index in patients with advanced or metastatic gastric cancer treated with immunotherapy. Nutrients. (2023) 15:4290. doi: 10.3390/nu15194290, PMID: 37836573 PMC10574242

[19] Kubota K Ito R Narita N Tanaka Y Furudate K Akiyama N. Utility of prognostic nutritional index and systemic immune-inflammation index in oral cancer treatment. BMC Cancer. (2022) 22:368. doi: 10.1186/s12885-022-09439-x, PMID: 35392843 PMC8991673

[20] Institute NC. Common Terminology Criteria for Adverse Events (CTCAE) Version 5.0 U.S. Department of Health and Human Services, National Institutes of Health, Bethesda, Maryland, USA: National Cancer Institute (2017).

[21] Hassanzad M Hajian-Tilaki K. Methods of determining optimal cut-point of diagnostic biomarkers with application of clinical data in ROC analysis: an update review. BMC Med Res Methodol. (2024) 24:84. doi: 10.1186/s12874-024-02198-2, PMID: 38589814 PMC11000303

[22] Sadatsafavi M Adibi A Puhan M Gershon A Aaron SD Sin DD. Moving beyond AUC: decision curve analysis for quantifying net benefit of risk prediction models. Eur Respir J. (2021) 58:2101186. doi: 10.1183/13993003.01186-2021, PMID: 34503984

[23] Nakasone Elizabeth S Askautrud Hanne A Kees T Park J-H Plaks V Ewald AJ. Imaging tumor-stroma interactions during chemotherapy reveals contributions of the microenvironment to resistance. Cancer Cell. (2012) 21:488–503. doi: 10.1016/j.ccr.2012.02.017 22516258 PMC3332002

[24] Liu X Wu Z Lin E Li W Chen Y Sun X. Systemic prognostic score and nomogram based on inflammatory, nutritional and tumor markers predict cancer-specific survival in stage II-III gastric cancer patients with adjuvant chemotherapy. Clin Nutr. (2019) 38:1853–60. doi: 10.1016/j.clnu.2018.07.015, PMID: 30075998

[25] Chen L Bai P Kong X Huang S Wang Z Wang X. Prognostic nutritional index (PNI) in patients with breast cancer treated with neoadjuvant chemotherapy as a useful prognostic indicator. Front Cell Dev Biol. (2021) 9:656741. doi: 10.3389/fcell.2021.656741, PMID: 33859986 PMC8042235

[26] Buzby GP Mullen JL Matthews DC Hobbs CL Rosato EF. Prognostic nutritional index in gastrointestinal surgery. Am J Surg. (1980) 139:160–7. doi: 10.1016/0002-9610(80)90246-9, PMID: 7350839

[27] Yang G Wang D He L Zhang G Yu J Chen Y. Normal reference intervals of prognostic nutritional index in healthy adults: a large multi-center observational study from Western China. J Clin Lab Anal. (2021) 35:e23830. doi: 10.1002/jcla.23830, PMID: 34018637 PMC8274996

[28] Bacalbasa N Petrea S Gaspar B Pop L Varlas V Hasegan A. The influence of inflammatory and nutritional status on the long-term outcomes in advanced stage ovarian cancer. Cancers (Basel). (2024) 16:2504. doi: 10.3390/cancers16142504, PMID: 39061143 PMC11274520

[29] Wang J Zhu R Fang H Xing X Ge L Cai G. Association of prognostic nutritional index with the presence and all-cause mortality of rheumatoid arthritis: the national health and nutrition examination survey 2003-2018. BMC Public Health. (2024) 24:3281. doi: 10.1186/s12889-024-20795-0, PMID: 39593001 PMC11590335

[30] Inoue DS Janini Gomes M. Integrative insights into PNI: low-grade chronic inflammation, skeletal muscle wasting, and brain impairments. Brain Behav Immun Health. (2024) 40:100838. doi: 10.1016/j.bbih.2024.100838, PMID: 39228969 PMC11369383

[31] Huang X Hu H Zhang W Shao Z. Prognostic value of prognostic nutritional index and systemic immune-inflammation index in patients with osteosarcoma. J Cell Physiol. (2019) 234:18408–14. doi: 10.1002/jcp.28476, PMID: 30891768

[32] D'Alessandro C Benedetti A Di Paolo A Giannese D Cupisti A. Interactions between food and drugs, and nutritional status in renal patients: a narrative review. Nutrients. (2022) 14:212. doi: 10.3390/nu14010212, PMID: 35011087 PMC8747252

[33] Ma C Yu R Li J Guo J Xu J Wang X. Preoperative prognostic nutritional index and systemic immune-inflammation index predict survival outcomes in osteosarcoma: a comparison between young and elderly patients. J Surg Oncol. (2022) 125:754–65. doi: 10.1002/jso.26757, PMID: 34811745

[34] Wang S Cowley LA Liu XS. Sex differences in cancer immunotherapy efficacy, biomarkers, and therapeutic strategy. Molecules. (2019) 24:3214. doi: 10.3390/molecules24183214, PMID: 31487832 PMC6767080

[35] Crawford J Dale DC Lyman GH. Chemotherapy-induced neutropenia: risks, consequences, and new directions for its management. Cancer. (2004) 100:228–37. doi: 10.1002/cncr.11882, PMID: 14716755

[36] Arends J Bachmann P Baracos V Barthelemy N Bertz H Bozzetti F. ESPEN guidelines on nutrition in cancer patients. Clin Nutr. (2017) 36:11–48. doi: 10.1016/j.clnu.2016.07.015, PMID: 27637832

[37] Thompson KL Elliott L Fuchs-Tarlovsky V Levin RM Voss AC Piemonte T. Oncology evidence-based nutrition practice guideline for adults. J Acad Nutr Diet. (2017) 117:297–310.e47. doi: 10.1016/j.jand.2016.05.010, PMID: 27436529

[38] Muscaritoli M Arends J Bachmann P Baracos V Barthelemy N Bertz H. ESPEN practical guideline: clinical nutrition in cancer. Clin Nutr. (2021) 40:2898–913. doi: 10.1016/j.clnu.2021.02.005, PMID: 33946039

