# Supplementary figures and images for: Association between Prognostic Nutritional Index and myelosuppression in gastric cancer patients undergoing chemotherapy: a retrospective cohort study
Source: Front Nutr. 2025 Oct 7;12:1605421. doi: 10.3389/fnut.2025.1605421 (PMC12537429; doi:10.3389/fnut.2025.1605421)

**PNI**  
**AUC = 0.729**

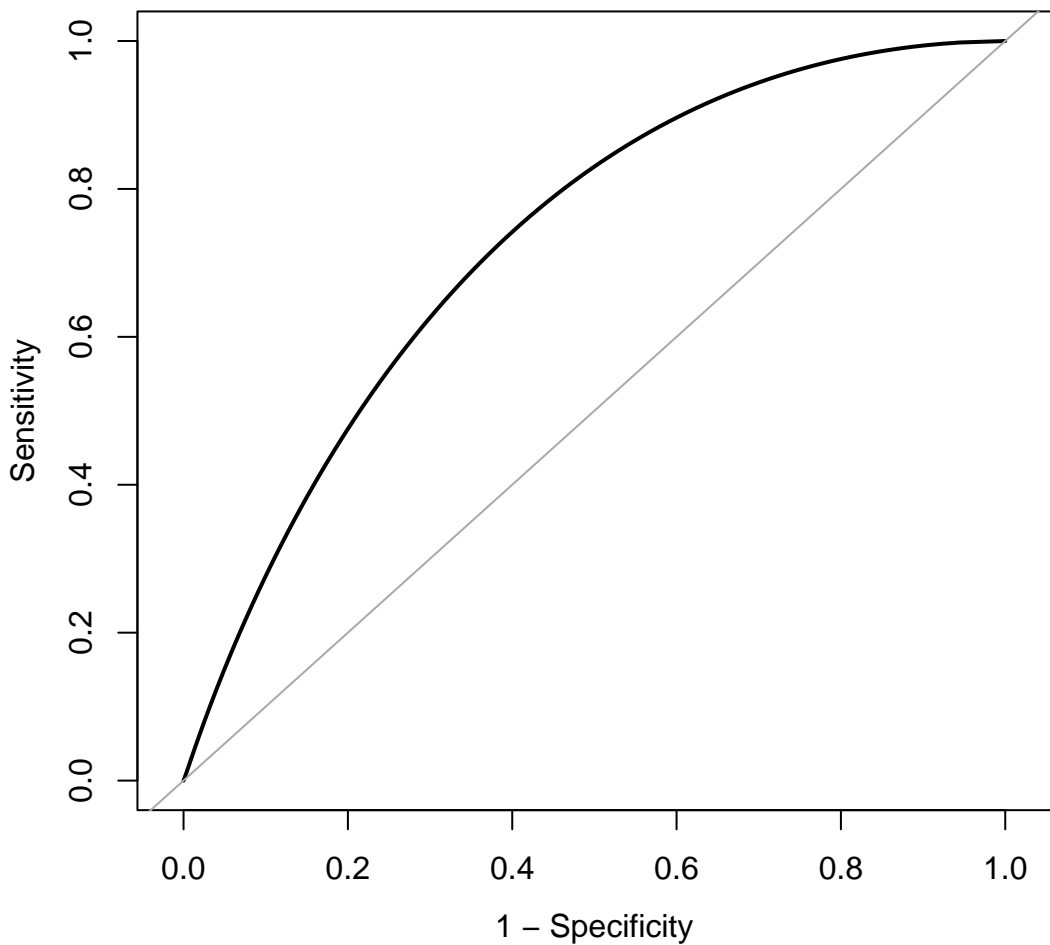

Supplement: SUPPLEMENTARY FIGURE 1 — The smooth ROC curve of PNI for predicting chemotherapy-induced myelosuppression in gastric cancer patients, obtained using the bootstrapping method (resample: 500), with an area under the receiver operating characteristic curve (ROC). [file Image_1.PDF]

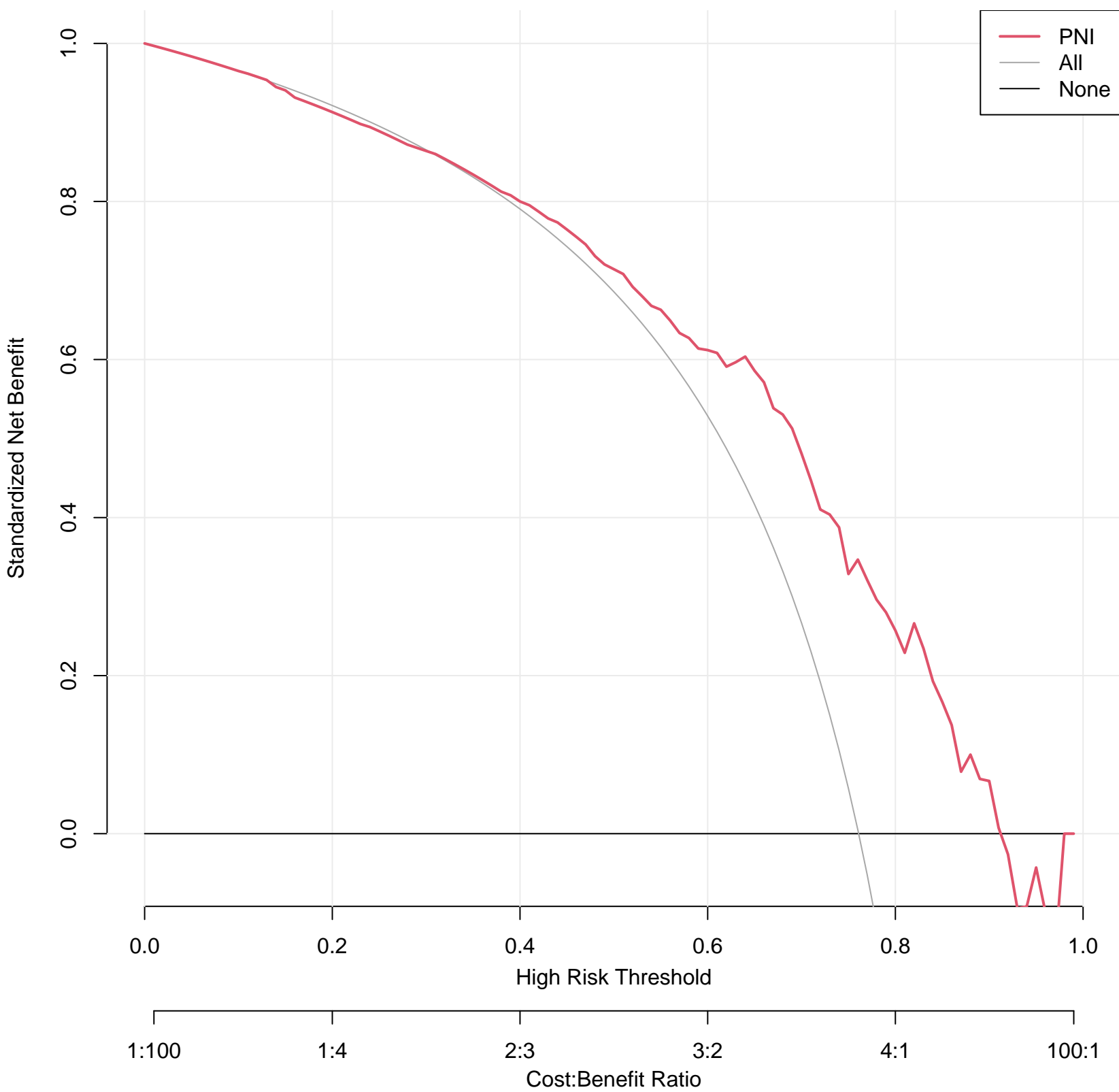

Supplement: SUPPLEMENTARY FIGURE 2 — The DCA curve of PNI for the prediction of chemotherapy-induced myelosuppression in gastric cancer patients. [file Image_2.PDF]
