# Supplementary material for: Association between Prognostic Nutritional Index and myelosuppression in gastric cancer patients undergoing chemotherapy: a retrospective cohort study
Source: Front Nutr. 2025 Oct 7;12:1605421. doi: 10.3389/fnut.2025.1605421 (PMC12537429; doi:10.3389/fnut.2025.1605421)
Supplement: Supplementary file 3 [file Table_1.DOCX]

**Supplementary table 1 PNI Threshold Analysis for Myelosuppression Diagnosis in Gastric Cancer**

| **Variable** | **Best Threshold** | **AUC** | **Specificity** | **Sensitivity** | **Accuracy** | **Positive-LR** | **Negative-LR** | **Diagnose-OR** | **Postive-pv** | **Negative-pv** |
| --- | --- | --- | --- | --- | --- | --- | --- | --- | --- | --- |
| PNI | 48.025 | 0.729 | 0.5152 | 0.8762 | 0.7899 | 1.8071 | 0.2403 | 7.5192 | 0.8519 | 0.5667 |

Notes: PNI=Prognostic Nutritional Index,AUC**=**Area Under Curve, Positive-LR=Positive Likelihood Ratio, Negative-LR=Negative Likelihood Ratio,

Diagnose-OR=Diagnostic Odds Ratio,PPV=Positive Predictive Value, NPV=Negative Predictive Value.
